# Bimetallic Metal-Organic Framework-Derived Carbon Nanotube-Based Frameworks for Enhanced Capacitive Deionization and Zn-Air Battery

**DOI:** 10.3389/fchem.2019.00449

**Published:** 2019-06-19

**Authors:** Wenhui Shi, Xilian Xu, Chenzeng Ye, Dongyong Sha, Ruilian Yin, Xuhai Shen, Xiaoyue Liu, Wenxian Liu, Jiangnan Shen, Xiehong Cao, Congjie Gao

**Affiliations:** ^1^Center for Membrane Separation and Water Science and Technology, Ocean College, Zhejiang University of Technology, Hangzhou, China; ^2^College of Materials Science and Engineering, Zhejiang University of Technology, Hangzhou, China

**Keywords:** metal-organic frameworks, carbon nanotubes, capacitive deionization, oxygen evolution reaction, Zn-air battery, flexible devices, three-dimensional graphene, hierarchical structures

## Abstract

Carbon-based materials have attracted intensive attentions for a wide range of energy and environment-related applications. Energy storage/conversion devices with improved performance have been achieved by utilization of metal-organic-framework (MOF)-derived carbon structures as active materials in recent years. However, the effects of MOF precursors on the performance of derived carbon materials are rarely investigated. Here, we report that the incorporation of small amount of Fe or Ni in Co-based MOFs leads to a significant enhancement for the derived carbon nanotube-based frameworks (CNTFs) in Na^+^/Cl^−^ ion electrosorption. Further investigation revealed the enhanced performance can be attributed to the improved specific surface area, electrical conductivity, and electrochemical activity. Notably, the CoFe-CNTF derived from bimetallic CoFe-MOFs achieves a high ion adsorption capacity of 37.0 mg g^−1^, superior to most of recently reported carbon-based materials. Furthermore, the CoFe-CNTF also demonstrates high catalytic activity toward oxygen evolution reaction (OER) with a Tafel slope of 87.7 mV dec^−1^. After combination with three-dimensional graphene foam (3DG), the resultant CoFe-CNTF-coated 3DG is used as air-cathode to fabricate a flexible all-solid-state Zn-air battery, which exhibits a high open circuit potential of 1.455 V. Importantly, the fabricated flexible battery can light a light-emitting diode (LED) even when it is bent. This work provides new insights into designs of high-performance and flexible electrode based on MOF-derived materials.

## Introduction

Metal-organic frameworks (MOFs), as a group of porous crystalline materials with tailorable composition and structure, are versatile precursors for carbon-based porous materials (Tang et al., 2015; Xu et al., 2017, 2018). MOF-derived carbon-based materials have attracted intensive attentions for a wide range of applications, benefiting from their high surface area and well-defined porosity (Wang et al., 2016, 2017b, 2019; Li et al., 2018b; Liu et al., 2019a). Capacitive deionization (CDI) has emerged as a potential water treatment technology owing to its low cost and energy consumption (Song et al., 2019; Xu et al., 2019). In the CDI process, upon the application of an external voltage, ions in the solution are adsorbed and stored in the electrodes by forming electrical double layers (Suss et al., 2015; Hassanvand et al., 2018). A series of carbon-based materials have been extensively studied as CDI electrodes, such as activated carbon (Wu et al., 2016; Tang et al., 2019), graphene (Cao et al., 2014; Shi et al., 2016; Liu et al., 2017a; Li et al., 2018a), mesoporous carbon (Tsouris et al., 2011; Wang et al., 2014; Gao et al., 2019b), carbon nanotubes (Nie et al., 2012; Liu et al., 2015) and their composites (Xu et al., 2016b; Huang et al., 2017; Hu et al., 2018). Recently, MOF-derived porous carbon has demonstrated great potential for CDI applications. However, MOF-derived carbon materials generally have poor electrical conductivity because of low crystallinity of carbon (Gao et al., 2018, 2019a). Furthermore, abundant micropores within MOF-derived carbon structures limit the diffusion of ions into the electrode, leading to unsatisfactory CDI performance (Ding et al., 2017; Shi et al., 2018, 2019a). Therefore, novel strategies are required to further improve electron and ion transport of MOF-derived carbons (Wang et al., 2017a; Shi et al., 2019b).

In addition, MOF-derived nanostructured carbons are also attractive noble-metal-free electrocatalysts, which are promising in oxygen evolution reaction (OER) and have been used as cathode materials for Zn-air batteries recently (Liu et al., 2018, 2019b; Zhang et al., 2018b; Fang et al., 2019; Guo et al., 2019). Although the bright prospects of MOF-derived carbon materials, their practical applications are still restricted. First, micropores within MOF-derived porous carbon materials contribute to the majority of specific surface area, which have limited diffusion rate for electrolyte ions. Second, the development of next-generation flexible energy storage/conversion devices requires electrode materials with robust structure and strong mechanical properties under deformation (Han et al., 2018; Lv et al., 2018; Peng et al., 2018; Zhu et al., 2018; Ji et al., 2019). However, MOF-derived carbons are normally in powder form, which is difficult to form a firm interaction within a flexible electrode. Third, previous reports have shown that metallic content within MOF-derived carbons play an essential role in their catalytic properties (Liu et al., 2017b; Zhang et al., 2018a; Zheng et al., 2019). The effects of bimetallic MOF precursors on the performance of derived carbon materials are rarely investigated. Therefore, it is still a challenge to develop hierarchically porous, flexible, high-ion/charge-transport-rate MOF-derived carbon-based materials for high-performance Zn-air battery.

In this work, unique carbon nanotube-based frameworks (CNTFs) were obtained by a facile annealing process of rationally designed bimetallic MOF crystals (i.e., CoFe and CoNi-MOFs) as precursors. By the incorporation of a small amount of Fe or Ni in Co-based frameworks, the obtained CoFe- and CoNi-CNTFs both exhibited improved specific surface area and electrical conductivity, as compared to those of Co-CNTF. Due to the hierarchically porous structure with abundant exposed active sites, high conductivity for fast electron transfer, and unique nanostructures with metal nanoparticle embedded in CNTs, the CoFe- and CoNi-CNTF electrodes exhibited remarkably enhanced Na^+^/Cl^−^ ions adsorption property. Furthermore, high catalytic activity toward oxygen evolution reaction (OER) was also achieved by CoFe-CNTF. As a proof-of-concept application, an air-cathode based on CoFe-CNTF coated 3D graphene foam (CoFe-CNTF@3DG) was fabricated, and then used for flexible all-solid-state Zn-air battery, which displayed a high open circuit potential of 1.455 V.

## Experimental Section

### Material Synthesis

Preparation of Co-MOF: Typically, 1.4553 g of Co(NO_3_)_2_·6H_2_O was dissolved in 50 mL of methanol, which was mixed with 50 mL of methanol containing 3.2860 g of 2-methylimidazole. The obtained mixture was stirred at room temperature for 12 h. The sample was collected by centrifugation and washed with methanol for several times, followed by drying at 60°C for 12 h in vacuum oven.

Preparation of bimetallic CoFe and CoNi-MOFs: CoFe-MOF was synthesized by using Co(NO_3_)_2_·6H_2_O and Fe(NO_3_)_3_·6H_2_O with a molar ratio of 9:1. Typically, 1.3097 g of Co(NO_3_)_2_·6H_2_O and 0.2019 g of Fe(NO_3_)_3_·6H_2_O were dissolved in 50 mL of methanol. Then, 50 mL of methanol dissolved with 3.2860 g of 2-methylimidazole was added into the above solution followed by continuously stirring for 12 h at room temperature. CoFe-MOF was collected by centrifugation and washed with methanol for several times, followed by drying at 60°C for 12 h in vacuum oven. CoNi-MOF was synthesized by a similar procedure using Co(NO_3_)_2_·6H_2_O and Ni(NO_3_)_2_·6H_2_O with a molar ratio of 9:1.

Preparation of CNTFs: The as-prepared MOF precursors were pyrolyzed in a tube furnace by a two-step annealing process. The MOF precursors were first heated to 500°C with a heating rate of 5°C min^−1^, followed by bubbling ethanol vapor using H_2_ for 30 min. Then, the samples were heated at 800°C for 1 h under N_2_ atmosphere, followed by immersing the sample in 1 M HCl for 12 h to obtain CNTFs.

Preparation of CoFe-CNTF@3DG: 3D graphene foam (3DG) was obtained by using our previously reported method (Cao et al., 2011). Then 3DG was treated with a concentrated HNO_3_ solution at 80°C for 4 h, followed by washing with deionized water to obtain hydrophilic 3DG. The 3DG was placed into the precursor solution for CoFe-MOF and reacted for 12 h at room temperature. The CoFe-MOF@3DG composite was obtained by washing with methanol and then drying at 60°C for 12 h. The CoFe-CNTF@3DG was obtained according to the aforementioned annealing procedures for CNTF samples.

### Material Characterization

The morphologies of the samples were investigated by field-emission scanning electron microscopy (FE-SEM, Hitachi SU-8010), equipped with energy-dispersive X-ray spectroscopy (EDX) and transmission electron microscopy (TEM, JEM-100CX II). The crystal structures were characterized by powder X-ray diffraction (XRD, PNAlytical X'Pert PRO) with Cu Kα radiation. Nitrogen adsorption/desorption measurements were carried out by Micromeritics ASAP 2020. The specific surface areas were calculated by the multipoint Brunauer-Emmett-Teller (BET) method.

### Electrosorption Measurements

Batch-mode CDI tests were conducted by a circulating system, which includes a CDI module, a peristaltic pump (Longer Pump, YZ-1515x), a conductivity meter (Leici, DDSJ-308F) and a source meter (Keithley, SMU-2,400) (Figure S1A). The CDI electrodes were fabricated by coating a mixture of active materials, carbon black and poly(vinylidene fluoride) (PVDF) with a mass ratio of 8:1:1 in n-methylpyrrolidone (NMP) solution onto graphite paper, followed by drying at 60°C for 12 h in vacuum oven. As shown in Figure S1B, the CDI module was assembled with a CDI electrode, cation exchange membrane (Hangzhou Grion Environmental Technology Co., Ltd., LEHeCM-1, Type 1), spacer, anion exchange membrane (Hangzhou Grion Environmental Technology Co., Ltd., LE-HeAM-I, Type 1) and another CDI electrode. The total volume of NaCl aqueous solution is maintained at 50 mL. The electrosorption performance was measured at a flow rate of 30 mL min^−1^, varied applied voltages (1.0–1.6 V) and concentrations of NaCl solution (125–1,000 mg L^−1^). The electrosorption capacity, Γ (mg g^−1^), is calculated based on the following equation:

$$\begin{array}{l} \Gamma =\frac{\left({\text{C}}_{0}-{\text{C}}_{\text{t}}\right)V}{\text{m}} \end{array}$$

where C_0_ is the initial concentration of NaCl solution (mg L^−1^), C_t_ is the final concentration (mg L^−1^), V is the total volume of the solution (L), and m is the total mass of the electrodes (g).

### Electrochemical Measurements

The electrochemical properties were measured by an electrochemical workstation (Chenhua, China, CHI760E) in a three-electrode system at room temperature, using a carbon paper coated with prepared catalyst as the working electrode, a graphite rod as the counter electrode, a Saturated Calomel Electrode (SCE) as the reference electrode and 1.0 M KOH as the electrolyte. The catalyst slurry was obtained by adding 5 mg of CoFe-CNTF powder into a mixed solution of 768 μL DI water, 200 μL ethanol and 32 μL nafion, followed by ultrasonication for 30 min. The electrode was fabricated by coating the obtained slurry onto carbon paper with a loading mass of 1 mg cm^−2^. The Linear Sweep Voltammetry (LSV) was tested at a scan rate of 5 mV s^−1^ with a potential range from 1.2 to 1.7 V vs. Reversible Hydrogen Electrode (RHE). The Electrochemical Impedance Spectroscopy (EIS) measurement was conducted by an electrochemical station (Autolab PGSTAT302N) at a potential of 1.33 V vs. RHE in a frequency range from 100 kHz to 0.01 Hz with an amplitude of 5 mV.

### Fabrication of Zn-Air Battery

Zn-air battery was assembled using a carbon paper loaded with CoFe-CNTF catalyst as the air cathode, Zn plate as the anode, and a mixed solution of 6 M KOH and 0.2 M Zn(Ac)_2_ as the electrolyte, which was tested on a battery testing system (Land CT2001A). The flexible solid-state Zn-air battery was assembled by using CoFe-CNTF@3DG as the air electrode, nickel foam as the current collector, Zn plate as the anode, and polyacrylic acid (PAA) gel containing 11.25 M KOH and 0.25 M ZnO as the solid electrolyte.

## Results and Discussion

**Scheme 1:**
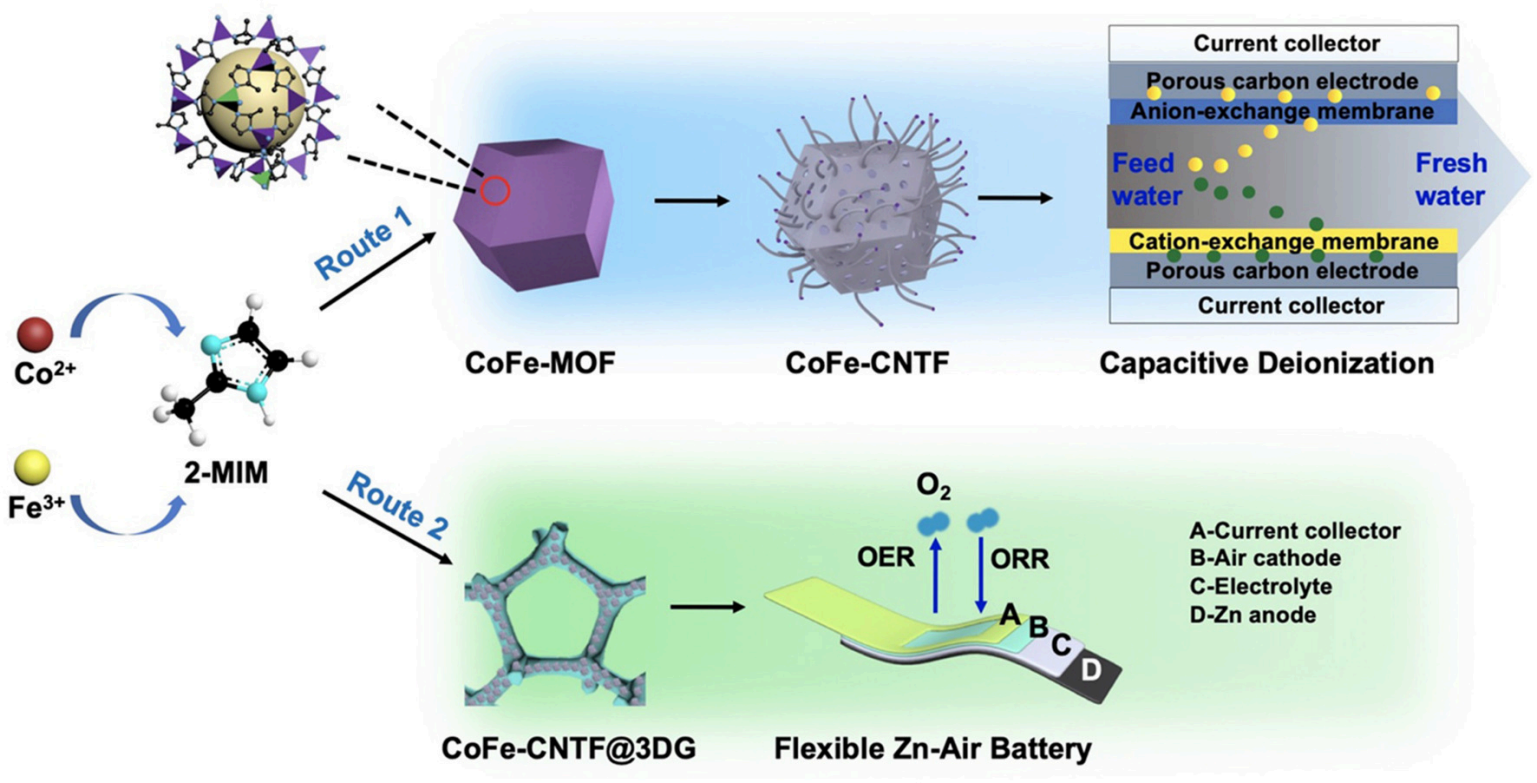
Schematic illustration of the synthetic processes of CoFe-CNTF and CoFe-CNTF@3DG as well as their applications for CDI and Zn-air battery.

The preparation of CNTF is schematically shown in Scheme 1. Bimetallic MOF crystals (i.e., CoFe- and CoNi-MOFs) were first synthesized using the mixtures of metal salts with a proper molar ratio (route 1, see Experimental Section). For comparison, monometallic Co-MOF was also prepared. Then, the as-prepared MOF precursors were undergone a two-step annealing process for pyrolysis of MOFs and catalytic growth of CNTs, followed by an acid treatment to obtain the CNT-based frameworks (i.e., CoFe-CNTF, CoNi-CNTF, and Co-CNTF). Furthermore, CoFe-MOFs were *in-situ* grown on 3D graphene foam (3DG) followed by similar annealing process to produce a flexible air cathode (CoFe-CNTF@3DG, route 2, see Experimental Section).

**Figure 1:**
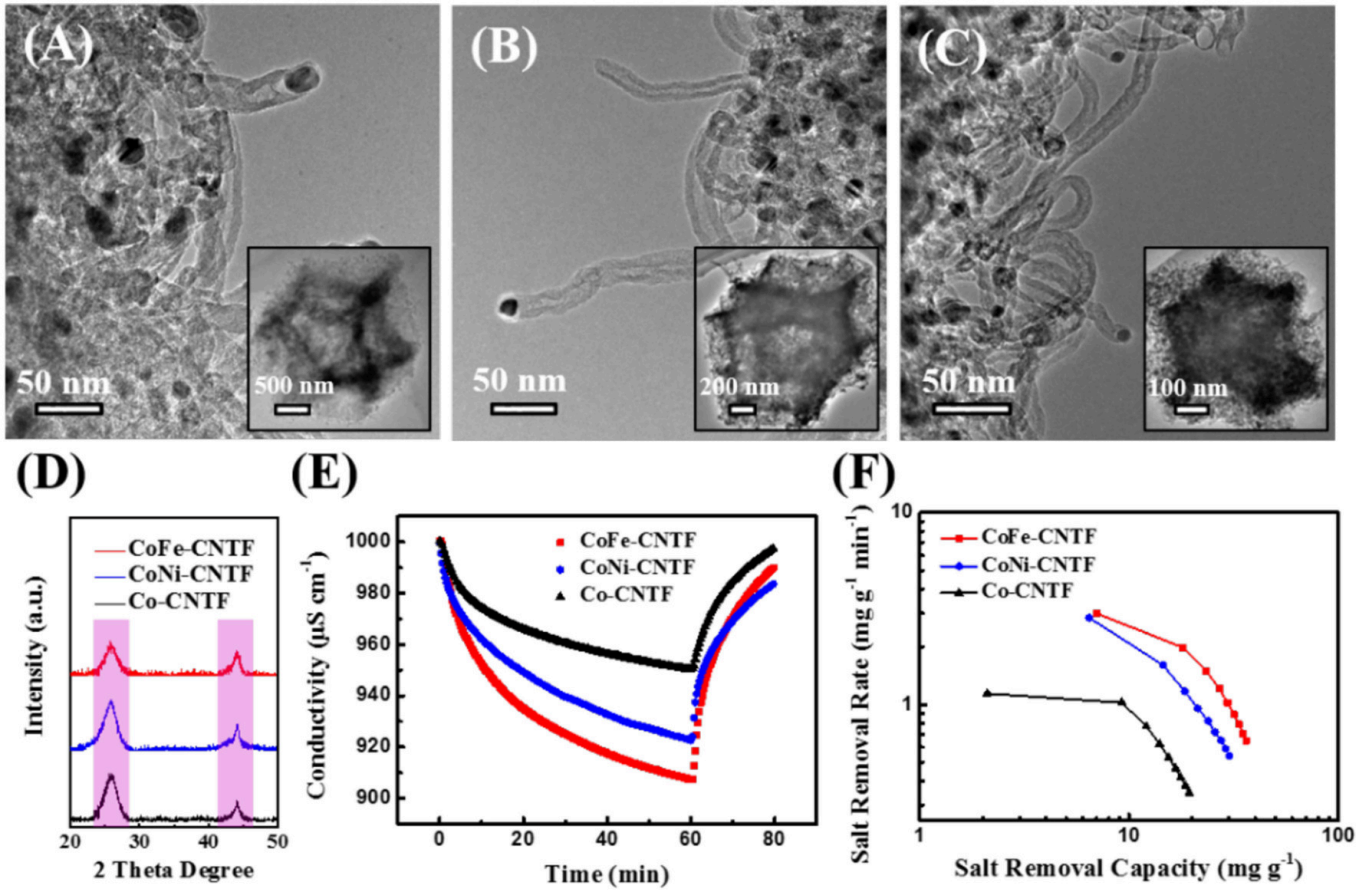
TEM images of **(A)** CoFe-CNTF, **(B)** CoNi-CNTF, and **(C)** Co-CNTF. Insets in **(A–C)**: corresponding low-magnification TEM images. **(D)** XRD patterns of CoFe-CNTF, CoNi-CNTF, and Co-CNTF. **(E)** Electrosorption behaviors and **(F)** CDI Ragone plots of CoFe-CNTF, CoNi-CNTF, and Co-CNTF in 500 mg L^−1^ NaCl solution at 1.2 V.

The scanning electron microscopy (SEM) images of prepared bimetallic MOF crystals exhibit typical dodecahedron shape (Figures S2A–C), which is similar with that of Co-MOF. In addition, the energy-dispersive X-ray spectroscopy (EDX) spectra in Figures S2D–F indicate all of the MOFs show signals of C, N, O, and Co. Besides, CoFe- and CoNi-MOFs contain additional Fe or Ni elements, suggesting the successful incorporation of Fe/Ni in the Co-based frameworks. The X-ray diffraction (XRD) patterns in Figure S3 show the same diffraction peaks for CoFe-, CoNi-MOFs, and Co-MOF, demonstrating the same crystal structure (Kaur et al., 2016). SEM images in Figure S4 further reveal that CNTFs derived from CoFe, CoNi, and Co-MOFs maintained a similar dodecahedron shape after the annealing process, which consist of interconnected CNT frameworks. Further investigations on the morphology and microstructure of CNTFs were performed by transmission electron microscopy (TEM). As shown in Figures 1A–C, the obtained CNTFs exhibited hierarchically porous structures assembled by CNTs, which have nanoparticles with diameters of 10–18 nm encapsulated on the top of tubes (Li et al., 2016). XRD patterns of all CNTFs exhibit similar characteristic peaks located at ~26 and 44° (Figure 1D), which can be assigned to the (002) and (101) planes of graphitic carbon (JCPDS: 41-1487) (Xu et al., 2016a).

The electrosorption performance of the prepared three CNTFs samples for Na^+^/Cl^−^ ions were evaluated by measuring change of solution conductivity at applied voltages using a CDI device (Figure S1, see experimental section). As displayed in Figure 1E, when an external voltage was applied, Na^+^/Cl^−^ ions were adsorbed and trapped in the electrodes, along with the gradually decreased solution conductivity. The NaCl electrosorption capacity of the CoFe-CNTF electrode is 37.0 mg g^−1^, which is much higher than that of Co-CNTF (19.9 mg g^−1^). Figure 1F depicts CDI Ragone plot of the three electrodes, in which the adsorption capacity and rate for Na^+^/Cl^−^ ions are used to assess the electrosorption behavior of the electrodes. Notably, in the CDI Ragone plot, the curve of CoFe-CNTF electrode is located in the upper right corner, suggesting both the highest Na^+^/Cl^−^ ions adsorption capacity and adsorption rate (Lee et al., 2018).

**Figure 2:**
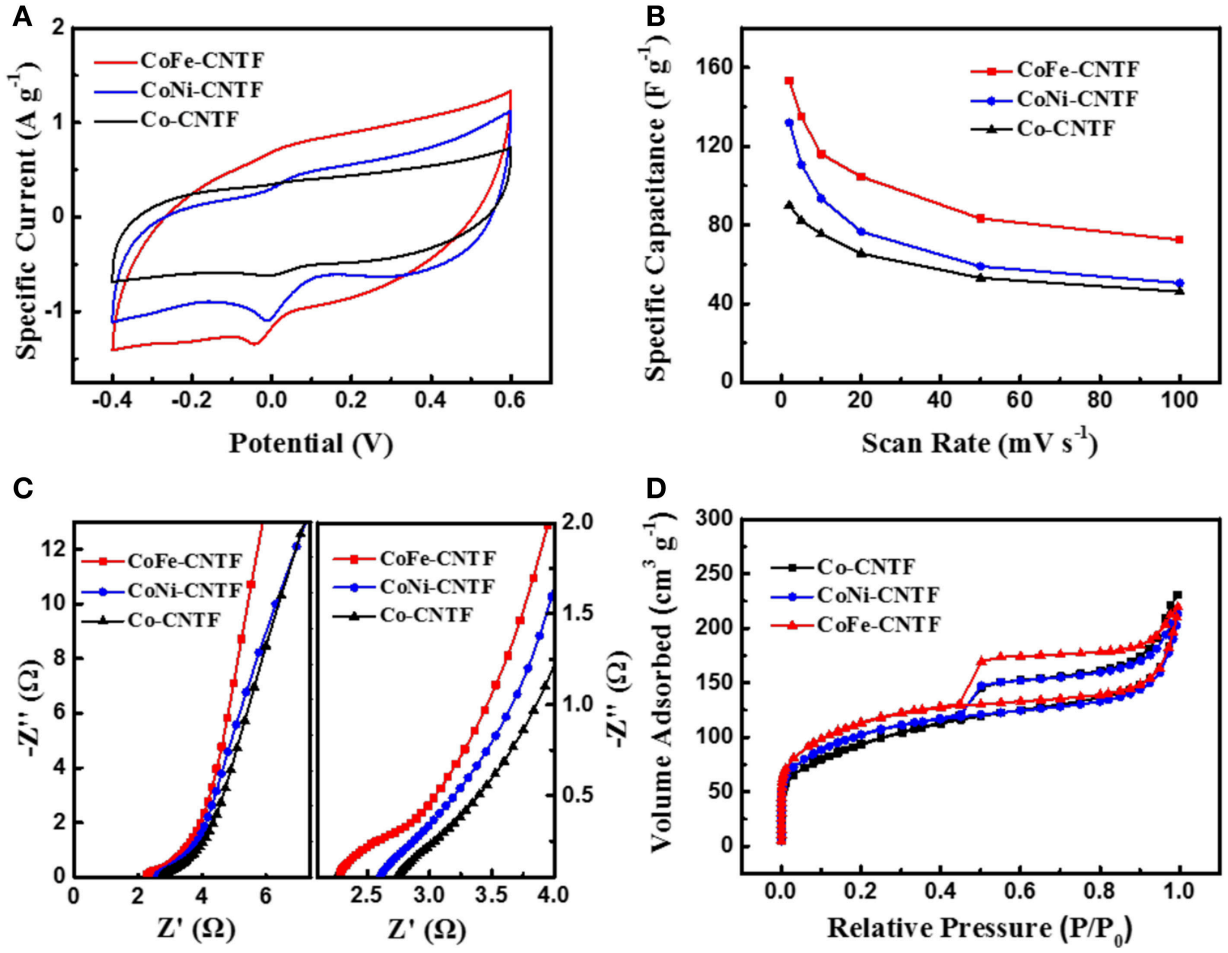
**(A)** CV curves of CoFe-CNTF, CoNi-CNTF, and Co-CNTF electrodes at a scan rate of 5 mV s^−1^. **(B)** Specific capacitances of CoFe-CNTF, CoNi-CNTF, and Co-CNTF at different scan rates. **(C)** Nyquist plots and **(D)** Nitrogen adsorption-desorption isotherms of CoFe-CNTF, CoNi-CNTF, and Co-CNTF.

The aforementioned results demonstrate that the remarkably enhanced Na^+^/Cl^−^ ions adsorption performance was achieved by the bimetallic MOF-derived CNTFs. To further reveal the origins of the improvement of CDI performance, further investigations on the properties of samples in terms of electrochemical capacitance, electrical conductivity, and specific surface area were carried out. The cyclic voltammetry (CV) profiles of CoFe-, CoNi-, and Co-CNTF electrodes are shown in Figure 2A. Obviously, the CoFe-CNTF electrode exhibited the largest integrated area of CV curve, which is consistent with the calculated specific capacitances. The CoFe-CNTF electrode showed the highest specific capacitance at various scan rates, i.e., 153 F g^−1^ at 2 mV s^−1^ and 72 F g^−1^ at 100 mV s^−1^ (see Figure 2B). To further study the electrical conductivity and internal resistivity of CNTF electrodes, electrochemical impedance spectroscopy (EIS) measurement was conducted. Figure 2C exhibits the Nyquist plots of CoFe-, CoNi-, and Co-CNTF electrodes in 1 M NaCl solution, which include a semicircle in the high frequency region and an inclined line in the low frequency region. The inclined line of the CoFe-CNTF electrode shows the largest slope, suggesting faster ion diffusion into the porous structure. In addition, the intercept of the curve in the high-frequency region with the real axis represents the equivalent series resistance (ESR) of the electrodes. The CoFe-CNTF electrode showed an ESR value of 2.38 Ω, smaller than that of Co-CNTF (2.83 Ω). The smaller ESR and fast ion diffusion of CoFe-CNTF electrode enhance the capacitive performance. Additionally, to investigate the specific surface area and pore structure of CNTFs, N_2_ adsorption-desorption measurements were carried out. As shown in Figure 2D, both CoFe- and CoNi-CNTFs displayed type-IV isotherms with sharply increased adsorption at low pressures and distinct hysteresis loops at medium pressure region, indicating existence of micropores, and abundant mesopores (Ding et al., 2018). Compared with CoFe- and CoNi-CNTFs, Co-CNTF exhibited a smaller hysteresis loop at high relative pressure, indicating that CoFe- and CoNi-CNTFs possesses more mesopores. Moreover, the pore size distributions of Co-CNTF, CoNi-CNTF, and CoFe-CNTF in Figure S5 also suggest more mesopores existed in CoNi-CNTF and CoFe-CNTF than that of Co-CNTF. The Brunauer-Emmett-Teller (BET) surface areas of CoFe and CoNi-CNTF are 410 and 369 m^2^ g^−1^, respectively, which is higher than that of Co-CNTF (334 m^2^ g^−1^). Obviously, the CoFe-CNTF with the highest specific surface area and abundant mesopore is in favor of the diffusion of ions, thus, it is promising electrode materials for CDI.

**Figure 3:**
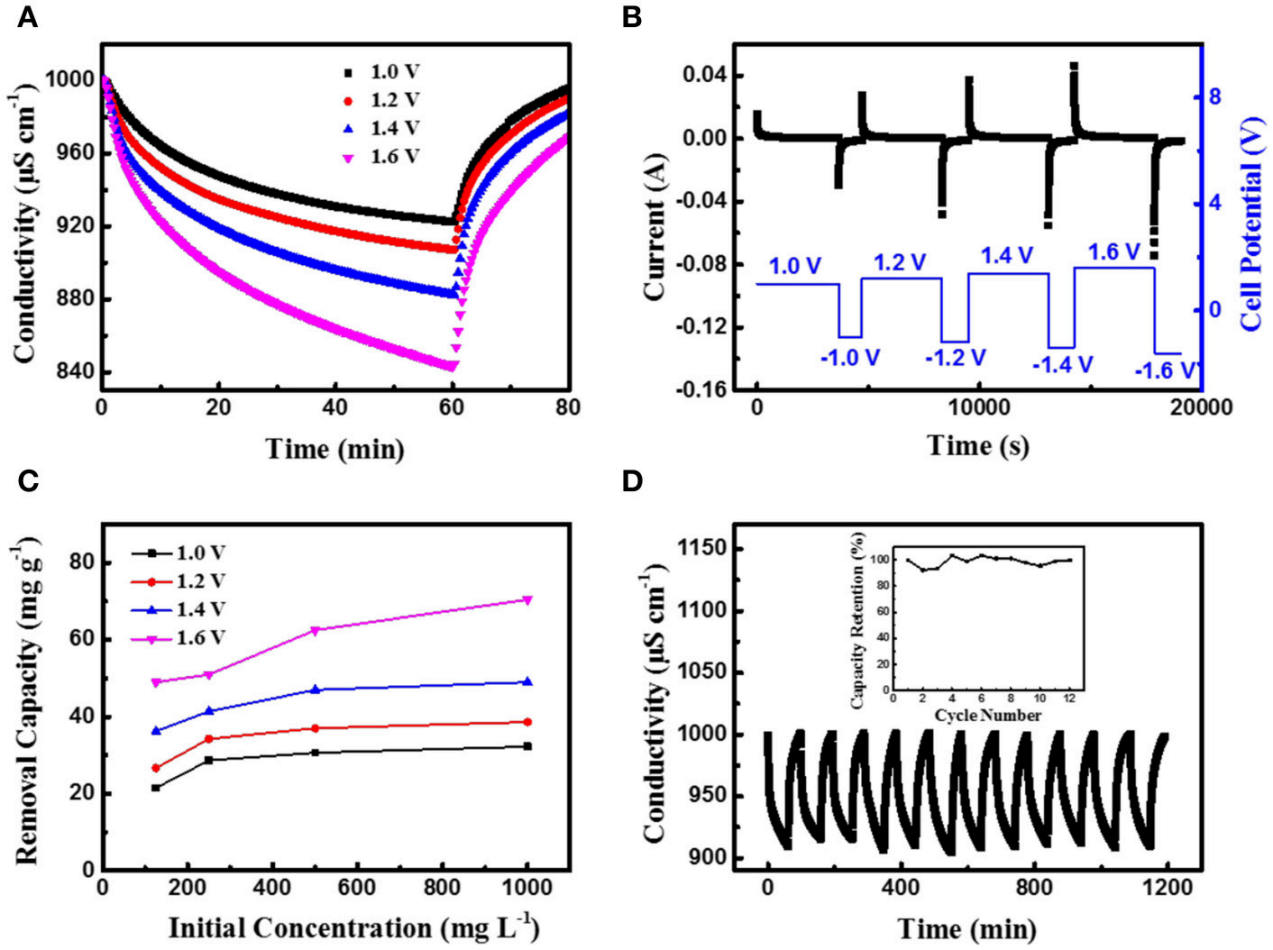
**(A)** Electrosorption behaviors and **(B)** the corresponding current curves of CoFe-CNTF in 500 mg L^−1^ NaCl solution at various cell voltages. **(C)** The electrosorption capacity of CoFe-CNTF at various cell voltages and concentrations of NaCl solution. **(D)** Cycling performance of CoFe-CNTF in 500 mg L^−1^ NaCl solution at 1.2 V. Inset is the capacity retention of CoFe-CNTF.

The CDI performance of the CoFe-CNTF electrode was further studied in terms of the effects of various applied voltages and concentrations of NaCl solution. As shown in Figure 3A, with the increase of the applied voltage, the electrosorption capacity was increased, indicating that a larger amount of ions were removed from the solution due to stronger electrostatic interaction. The corresponding transient current curves at different applied voltages in Figure 3B also show an increased current at higher voltages. Furthermore, electrosorption experiments in NaCl solutions of various initial concentrations ranging from 125 to 1,000 mg L^−1^ were carried out. Figure 3C demonstrates the calculated adsorption capacities of CoFe-CNTF electrode in different concentrations of NaCl solution at cell voltages of 1.0, 1.2, 1.4, and 1.6 V, respectively. With increment of the concentration of NaCl solution, the adsorption capacity increases as well. Notably, the CoFe-CNTF electrode exhibited a superior adsorption capacity of 37.0 mg g^−1^ in a 500 mg L^−1^ NaCl solution at 1.2 V, which is superior to most of the recently reported CDI electrode materials (see Table S1). Furthermore, the regeneration stability of the electrode was investigated by repeated electrosorption-desorption experiments at a voltage of 1.2 V. As shown in Figure 3D, CoFe-CNTF electrode exhibited excellent stability for 12 cycles without obvious capacity drop.

The above studies clearly demonstrate that CoFe-CNTF has an extraordinary specific surface area, conductivity, and electrochemical activity. This could be attributed to the hierarchically porous structure with abundant exposed active sites, high electrical conductivity for fast electron transfer, and unique nanostructures with Co/Fe nanoparticles embedded in CNTFs. These excellent properties endow them promising performance as air electrode of Zn-air batteries. As shown in Scheme 1, in order to apply it in flexible Zn-air batteries, the OER performance of CoFe-CNTF was first investigated. The OER activity of the as-obtained CoFe-CNTF was evaluated by a conventional three-electrode cell in 1.0 M KOH solution. Commercial IrO_2_ was also tested for comparison. As shown in Figure S6A, the CoFe-CNTF catalyst require a potential of 1.574 V vs. RHE (corresponding to overpotential of 344 mV) to achieve a current density of 10 mA cm^−2^, which is better than that of commercial IrO_2_ (1.658 V). Moreover, the Tafel slope of CoFe-CNTF is 87.7 mV dec^−1^, which is much smaller than that of commercial IrO_2_ (106.5 mV dec^−1^), suggesting faster OER catalytic kinetics of CoFe-CNTF (see Figure S6B). The Nyquist plots in Figure S6C demonstrate that the charge transfer resistance of CoFe-CNTF is much smaller than that of commercial IrO_2_, indicating that charge transfer process within CoFe-CNTF is more effective. Notably, CoFe-CNTF also exhibits excellent OER stability. As shown in Figure S6D, CoFe-CNTF shows nearly constant operating potential with a current density of 10 mA cm^−2^ for 12 h.

**Figure 4:**
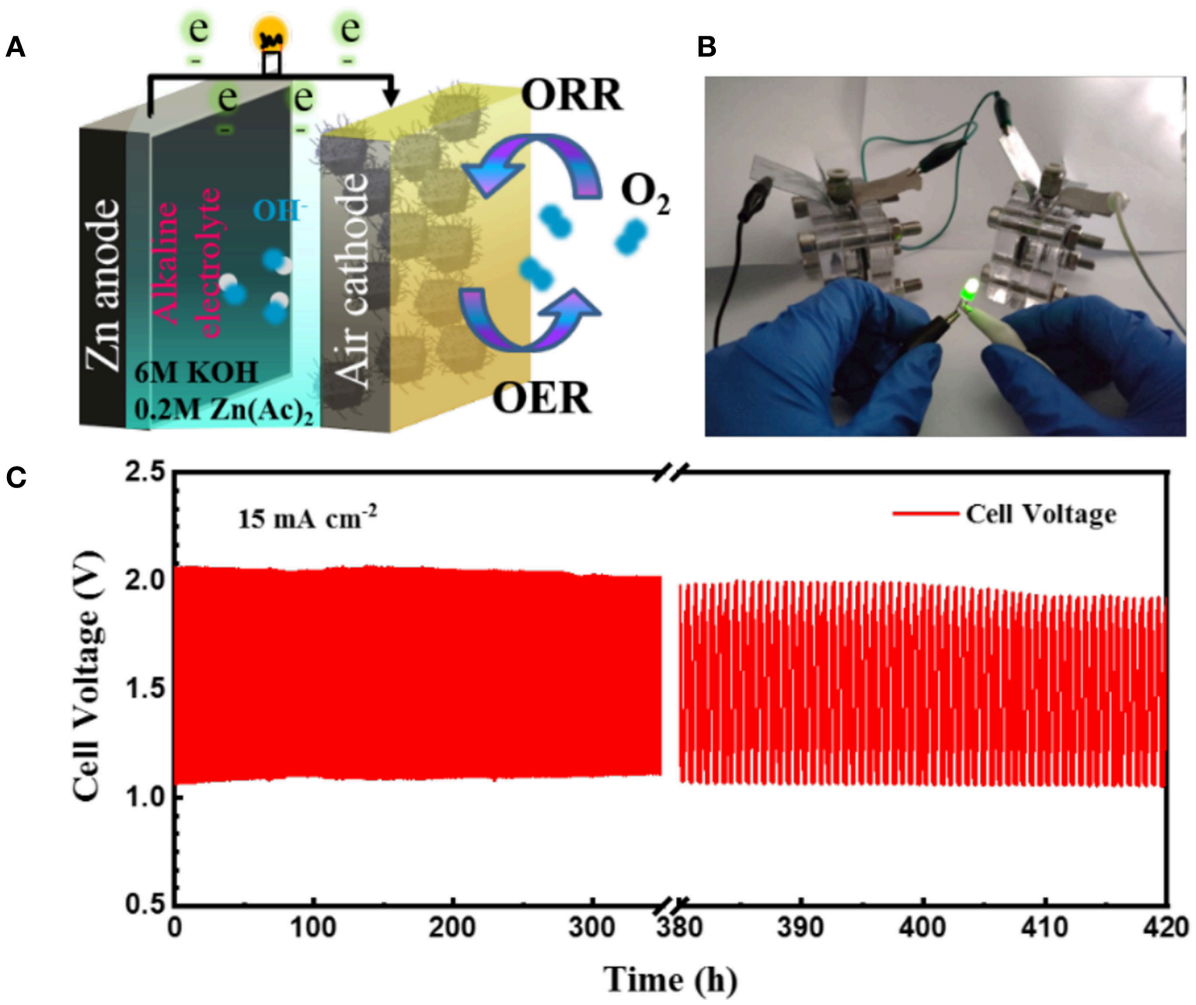
**(A)** Schematic illustration of Zn-air battery. **(B)** Photograph of the lightened LED bulb by two Zn-air batteries connected in series. **(C)** Discharge-charge cycling curves at a current density of 15 mA cm^−2^.

**Figure 5:**
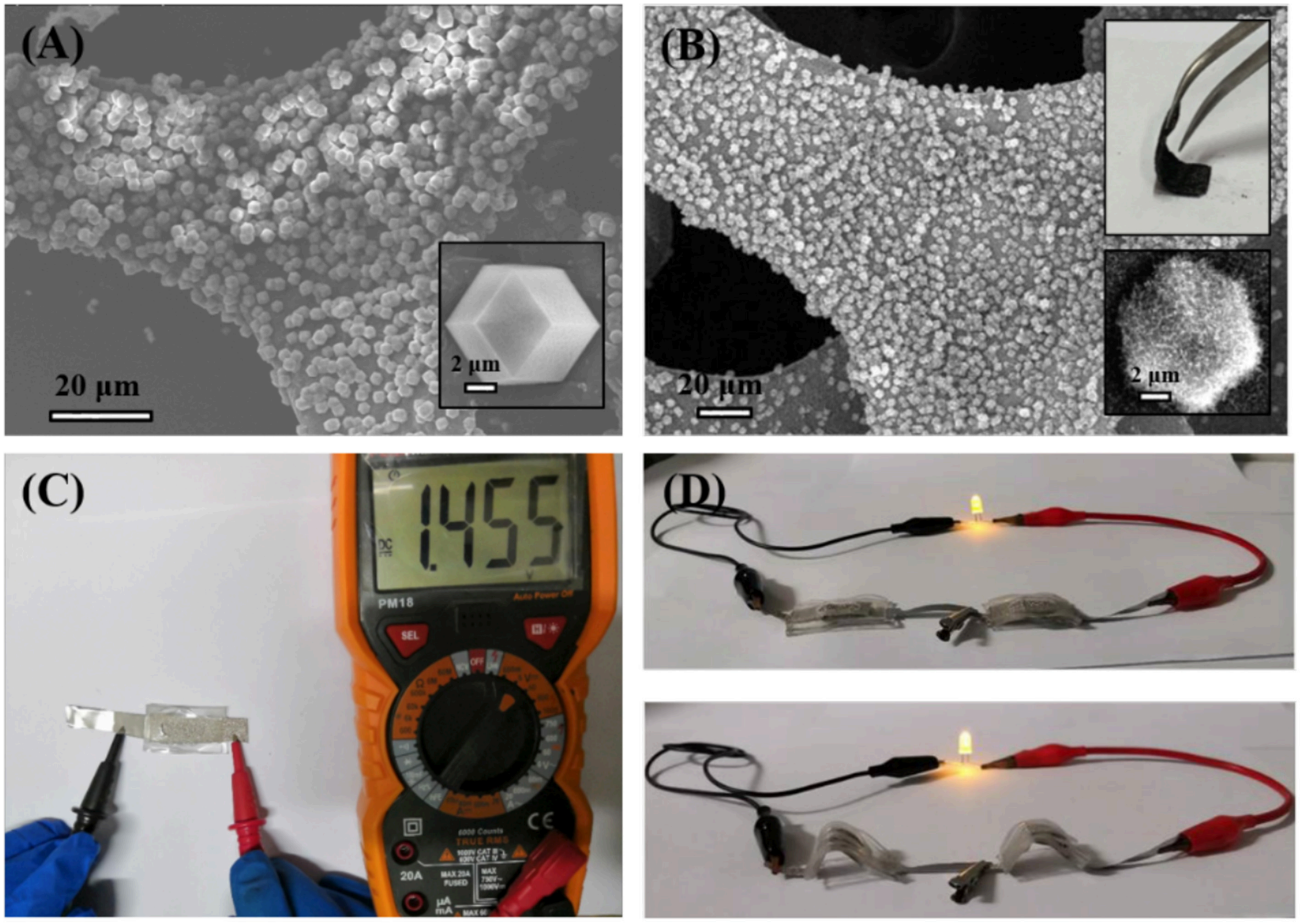
**(A)** SEM image of CoFe-MOF@3DG. Inset is high-magnification SEM image showing a CoFe-MOF crystal grown on the surface of 3DG. **(B)** SEM image of CoFe-CNTF@3DG. Insets: photograph of a bent CoFe-CNTF@3DG, and high-magnification SEM image (Bottom). **(C)** Open-circuit voltage of a fabricated all-solid-state Zn-air battery based on CoFe-CNTF@3DG. **(D)** Photographs of the lightened LED by two Zn-air batteries connected in series under different blending status.

To demonstrate the practical application of the obtained CoFe-CNTF, a rechargeable Zn-air battery was fabricated. Figure 4A shows a schematic diagram of the configuration of rechargeable Zn-air battery, in which CoFe-CNTF catalyst loaded on carbon paper was used as air-cathode, Zn plate as anode, and a mixed solution of 6 M KOH and 0.2 M Zn(Ac)_2_ as electrolyte. Two rechargeable Zn-air batteries connected in series can light up a light-emitting diode (LED) bulb successfully (Figure 4B). Furthermore, the fabricated Zn-air battery exhibited remarkable cycling stability after operation for 420 h at a current density of 15 mA cm^−2^ (Figure 4C). In addition, a flexible Zn-air battery based on CoFe-CNTF is also demonstrated. CoFe-MOFs were grown on 3DG (see Figure S7) followed by a similar annealing process to produce the flexible air cathode of CoFe-CNTF@3DG. The morphologies and XRD patterns of obtained CoFe-MOF@3DG and CoFe-CNTF@3DG are shown in Figures 5A,B and Figure S8. The photograph in the top inset of Figure 5B clearly demonstrates good flexibility of the obtained CoFe-CNTF@3DG electrode. The open-circuit voltage of the fabricated all-solid-state Zn-air battery is 1.455 V (Figure 5C). Importantly, a LED bulb could be successfully lightened by two Zn-air batteries connected in series under different blending status (Figure 5D), demonstrating its excellent flexibility and promising application in Zn-air batteries.

## Conclusion

In summary, we developed hierarchically porous carbon nanotube-based frameworks (CNTFs), which were derived from rationally designed bimetallic MOFs followed by a two-step annealing process. The CNTFs showed excellent Na^+^/Cl^−^ ion adsorption property. Especially, the CoFe-CNTF exhibited a remarkable adsorption capacity of 37.0 mg g^−1^ in a 500 mg L^−1^ NaCl solution at 1.2 V, superior to that of Co-CNTF (19.9 mg g^−1^). Such enhanced performance can be attributed to the larger surface area and improved electrical conductivity of the CoFe-CNTF. Moreover, due to the unique porous framework structure and abundant metal active sites, the CoFe-CNTF displayed excellent electrocatalytic performance in OER. CoFe-CNTF@3DG as air electrode demonstrated excellent flexibility and potential application for flexible all-solid-state Zn-air battery. We believe our work paves a way for the design of nanostructured carbon materials for the applications in sustainable energy conversion and storage.

## Data Availability

All datasets generated for this study are included in the manuscript and/or the Supplementary Files.

## Author Contributions

WS, XC, JS, CG, and WL designed the project. WS, CY, XS, and XL performed the synthesis, CDI experiment, and data analysis. XX, DS, and RY performed the Zn-air battery experiment and data analysis. All authors contribute to editing and discussion of the manuscript.

### Conflict of Interest Statement

The authors declare that the research was conducted in the absence of any commercial or financial relationships that could be construed as a potential conflict of interest.
