# Supplementary material for: Bimetallic Metal-Organic Framework-Derived Carbon Nanotube-Based Frameworks for Enhanced Capacitive Deionization and Zn-Air Battery
Source: Front Chem. 2019 Jun 19;7:449. doi: 10.3389/fchem.2019.00449 (PMC6593352; doi:10.3389/fchem.2019.00449)
Supplement: Supplementary file 1 [file Data_Sheet_1.pdf]

Supplementary Material

Bimetallic Metal-Organic Framework-derived Carbon Nanotube-based Frameworks for Enhanced Capacitive Deionization and Zn-air Battery

Wenhui Shi[1†], Xilian Xu[2†], Chenzeng Ye[1†], Dongyong Sha[2], Ruilian Yin[2], Xuhai Shen[2], Xiaoyue Liu[1], Wenxian Liu[2], Jiangnan Shen[1*], Xiehong Cao[2*], Congjie Gao[1]

[1]Center for Membrane Separation and Water Science & Technology, Ocean College, Zhejiang University of Technology, Hangzhou, China

[2]College of Materials Science and Engineering, Zhejiang University of Technology, Hangzhou, China

*** Correspondence:**
Jiangnan Shen
shenjn@zjut.edu.cn
Xiehong Cao
gcscaoxh@zjut.edu.cn

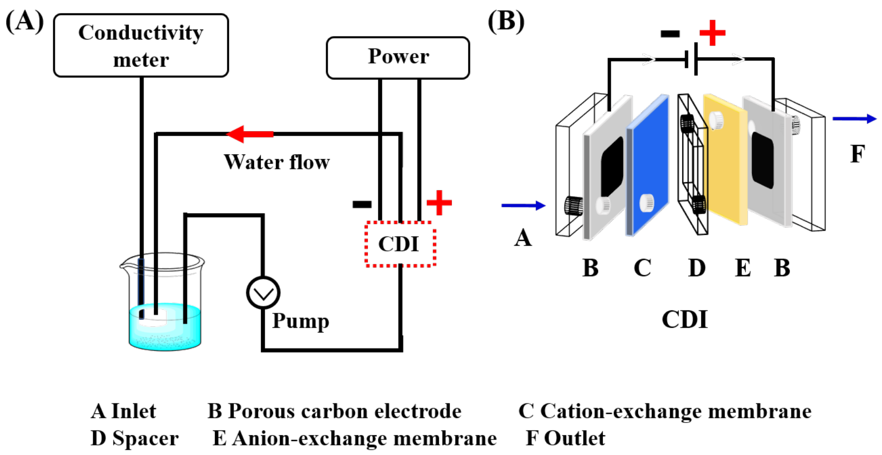

FIGURE S1 | Scheme for the experimental setups of **(A)** CDI process and **(B)** the CDI cell.

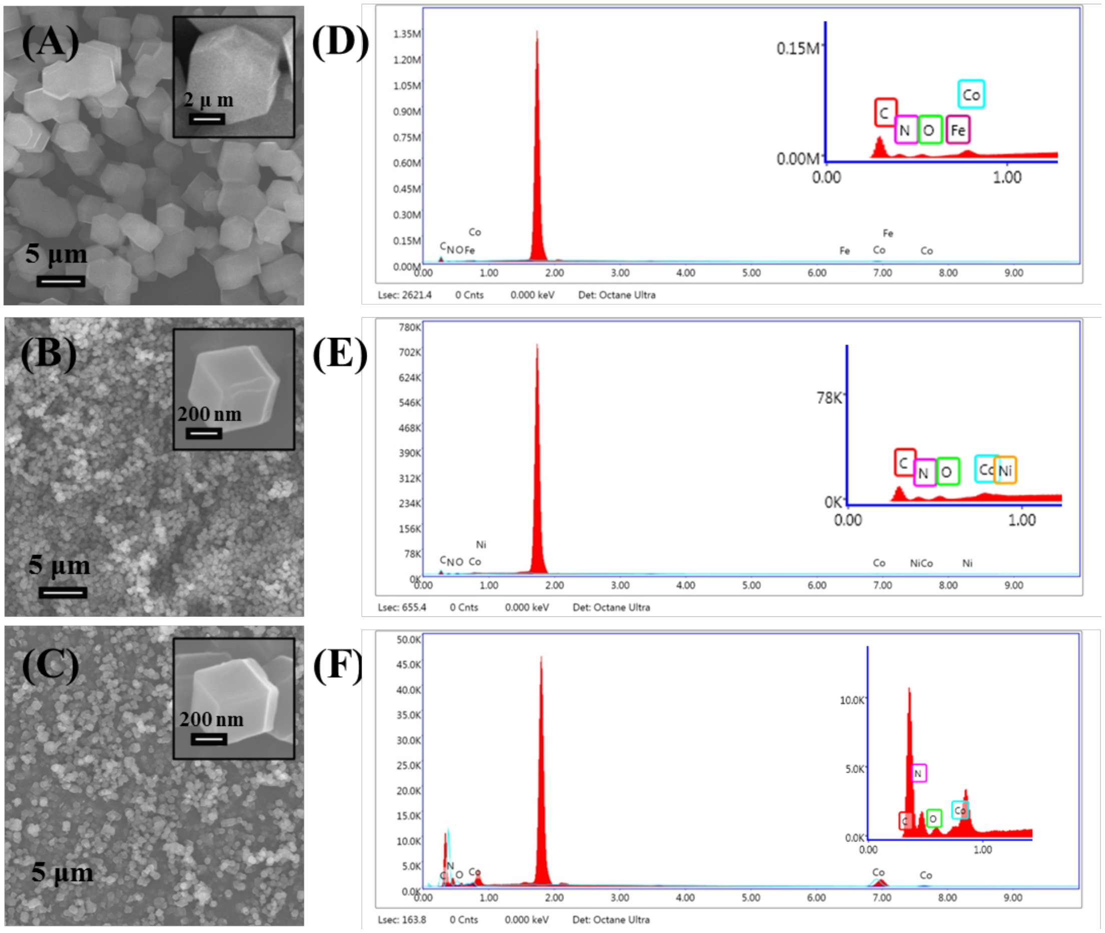

FIGURE S2 | **(A)-(C)** SEM images of CoFe-MOF, CoNi-MOF and Co-MOF crystals. **(D)-(F)** The corresponding EDX spectra of CoFe-MOF, CoNi-MOF and Co-MOF crystals.

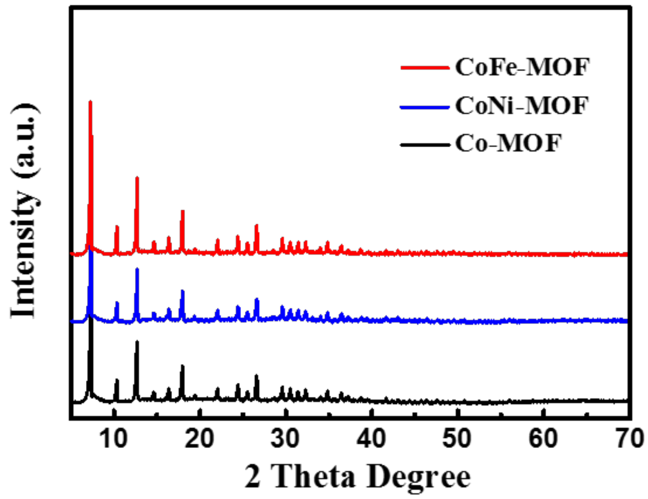

FIGURE S3 | XRD patterns of CoFe-MOF, CoNi-MOF and Co-MOF crystals.

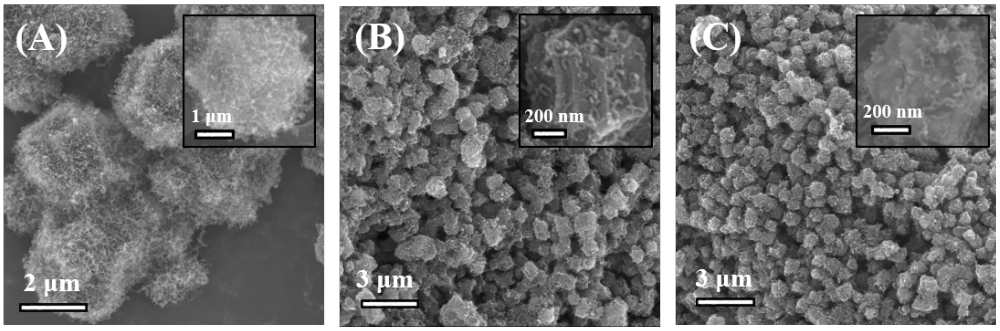

FIGURE S4 丨 SEM images of **(A)** CoFe-CNTF, **(B)** CoNi-CNTF and **(C)** Co-CNTF.

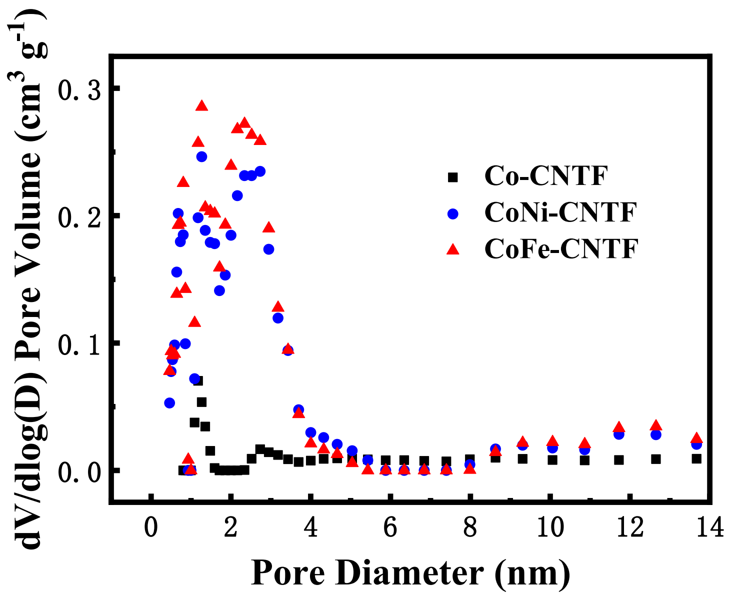

FIGURE S5 | The pore size distributions of Co-CNTF, CoNi-CNTF and CoFe-CNTF

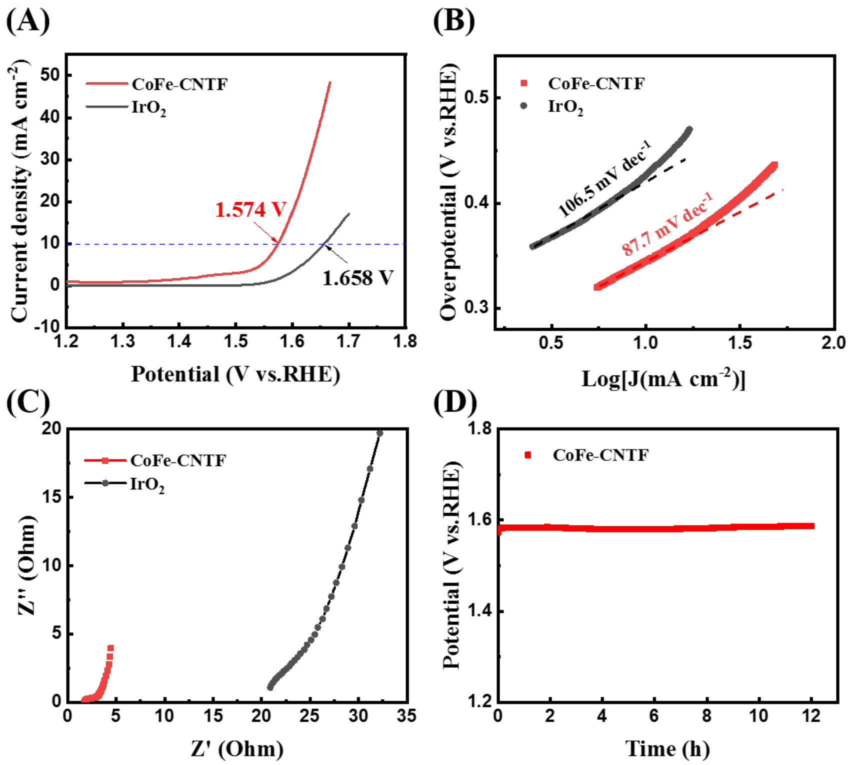

FIGURE S6 | (A) Linear sweep voltammetry (LSV) curves and **(B)** corresponding Tafel plots of CoFe-CNTF and IrO_2 in 1.0 M KOH at a scan rate of 5 mV s^{-1}. **(C)** Nyquist plots of CoFe-CNTF and IrO_2. **(D)** Galvanostatic measurement of OER of CoFe-CNTF at a constant current density of 10 mA cm^{-2}.

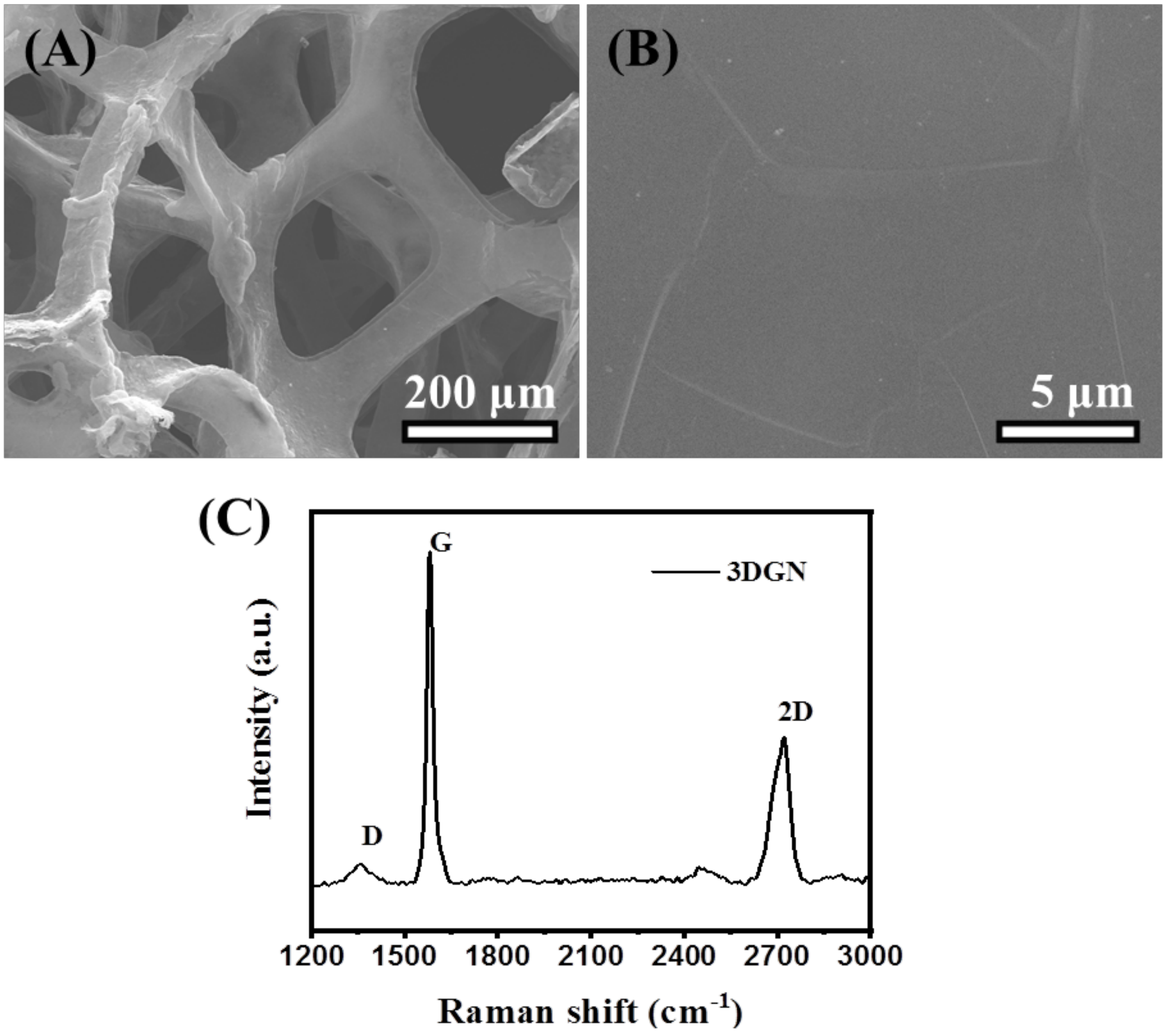

FIGURE S7 | (A, B) SEM images and (C) Raman spectrum of 3DG.

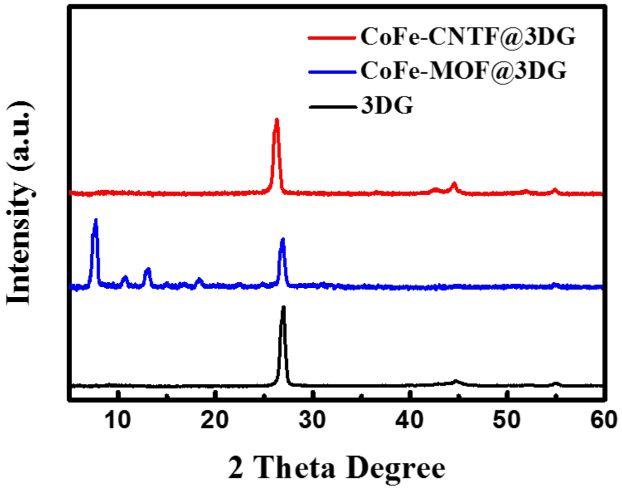

FIGURE S8 | XRD patterns of CoFe-CNTF@3DG, CoFe-MOF@3DG and 3DG.

Table S1. The comparison of CDI performance of our work with various recently reported carbon-based materials.

Electrode Materials	Cell Potential (V)	Initial Salt Concentration (mg L^{-1})	Removal Capacity (mg g^{-1})	Reference
TiO_2/GO	0.8	300	9.1	*Desalination*, 2015, 361, 53-64.
Graphene Laminate	1.2	400	15.3	*Desalination*, 2015, 357, 178-188.
Sulfonated Graphene-CNF	1.2	400	9.54	*Adv. Mater. Inter.*, 2015, 2, 1500372.
Nitrogen-doped graphene Sponge	1.2	500	14.8	*ACS Sustain. Chem. Eng.*, 2017, 5, 325-333.
PVA/Active Carbon	1.2	565	14.4	*Environ. Sci. Technol.*, 2018, 52, 5859-5867.
MIL-88-derived PC/rGO	1.2	500	30.3	*ACS Omega*, 2018, 3, 8506-8513.
CNFH	1.4	500	43.3	*Adv. Mater. Technol.*, 2018, 1800135.
Bimetallic MOF derived PC	1.4	750	45.62	*J. Mater. Chem. A*, 2017, 5, 6113-6121.
Co-Fe-CNT/PC	1.2	500	37.0	**This work**
	1.4	500	47.0	
